# Dr. Allport’s Pyorrhœa Instruments

**Published:** 1886-01

**Authors:** 


### DR. ALLPORT'S PYORRHŒA INSTRUMENTS.

One of the most complete sets of instruments of any kind, that we have yet seen, is that of Dr. W. W. Allport, for the treatment of pyorrhœa. Devised for his own use, we do not know that they are to be found in the dental depots, although Dr. Allport could doubtless have them made for any one who wishes. The set comprises thirteen beautiful scrapers, for both the pulling and pushing motion, besides an explorer and half a dozen bars expressly designed for dressing the edge of the alveolar border, without injury to the gums or teeth. There is no exposed point, on either the teeth or their investment, that cannot be easily reached with one of these instruments, which are all contained in a neat rosewood case. Equipped with this set, the operator is prepared for the operative treatment of any case of pyorrhœa that he may meet.

